# Elevated luteinizing hormone receptor signaling or selenium treatment leads to comparable changes in adrenal cortex histology and androgen-AR/ZIP9 signaling

**DOI:** 10.1007/s00709-023-01910-1

**Published:** 2023-12-06

**Authors:** Jaroslaw Wieczorek, Piotr Pawlicki, Marta Zarzycka, Laura Pardyak, Piotr Niedbala, Michal Duliban, Begum Yurdakok-Dikmen, Malgorzata Kotula-Balak

**Affiliations:** 1 Present Address: University Centre of Veterinary Medicine JU-UA, University of Agriculture in Krakow, Mickiewicza 24/28, 30-059, Krakow, Poland; 2 Present Address: Department of Medical Biochemistry, Jagiellonian University Medical College, Krakow, Poland; 3 Center of Experimental and Innovative Medicine, University of Agriculture in Kraków, 30-248 Krakow, Poland; 4 Department of Genetics, Animal Breeding and Ethology, Faculty of Animal Science, University of Agriculture in Krakow, Mickiewicza 24/28, 30-059 Krakow, Poland; 5 Department of Endocrinology, Institute of Zoology, Jagiellonian University in Krakow, Gronostajowa 9, 30-387, Krakow, Poland; 6 Department of Pharmacology and Toxicology, Ankara University Faculty of Veterinary Medicine, Dışkapı, 06110 Ankara, Turkey

**Keywords:** Adrenals, Androgens, Androgen receptor, Luteinizing hormone receptor, Selenium, zinc transporter

## Abstract

The importance and regulation of adrenal androgen production and signaling are not completely understood and are scarcely studied. In addition, there is still a search for appropriate animal models and experimental systems for the investigation of adrenal physiology and disease. Therefore, the main objective of the study was to evaluate the effect of luteinizing hormone (LH) signaling and selenium (Se2+) exposure on androgen adrenal signaling *via* canonical androgen receptor (AR), and membrane androgen receptor acting as zinc transporter (zinc- and iron-like protein 9; ZIP9). For herein evaluations, adrenals isolated from transgenic mice with elevated LH receptor signaling (KiLHRD^582G^) and adrenals obtained from rabbits used for *ex vivo* adenal cortex culture and exposure to Se2+ were utilized. Tissues were assessed for morphological, morphometric, and Western blot analyses and testosterone and zinc level measurements.

Comparison of adrenal cortex histology and morphometric analysis in KiLHRD^582G^ mice and Se2+-treated rabbits revealed cell hypertrophy. No changes in the expression of proliferating cell nuclear antigen (PCNA) were found. In addition, AR expression was decreased (*p* < 0.001) in both KiLHRD^582G^ mouse and Se2+-treated rabbit adrenal cortex while expression of ZIP9 showed diverse changes. Its expression was increased (*P* < 0.001) in KiLHRD^582G^ mice and decreased (*P* < 0.001) in Se2+-treated rabbits but only at the dose 10 ug/100 mg/ tissue. Moreover, increased testosterone levels (*P* < 0.05) and zinc levels were detected in the adrenal cortex of KiLHRD^582G^ mice whereas in rabbit adrenal cortex treated with Se2+, the effect was the opposite (*P* < 0.001).

## Introduction

In 1856, Charles E. Brown-S´equard demonstrated that bilateral adrenalectomy results in rapid death (Brown-S´equard 1856). In the early twentieth century, adrenal hormones, catecholamines, and corticosteroids were isolated and synthesized by Edward C. Kendall, Tadeusz Reichstein, and Philip Showalter Hench who were awarded the Nobel Prize in Physiology or Medicine in 1950. Since that time, adrenal endocrine functions and dysfunctions have been the subjects of research attempts.

The mammalian adrenal cortex is a source of steroid hormones (progestins, mineralocorticoids, glucocorticoids, androgens, and estrogens). Based on the cellular morphological criteria in humans and primates, the adrenal cortex is composed of steroidogenic cells. It is divided into three zones: *zona glomerulosa* (ZG), *zona fasciculata* (ZF), and *zona reticularis* (ZR) which additionally play unique functions being subjected to specific molecular characteristics and regulatory mechanisms (Arnold 1866). In the adrenal cortex of mice, ZF and neighboring the medulla X zone are present (Peaker 2019). The latter zone is homologous with the human fetal adrenal cortex (which is substituted with ZF after puberty in male mice) or with the juxtamedullary zone which exists in rabbits (Chester Jones 1957). In ZF because ZR is not separated, in response to luteinizing hormone (LH) stimulation, small amounts of androgens are produced having a pleiotropic effect as prohormones (dehydroepiandrosterone; DHEA, its sulfate derivatives; DHEAS, androstendione, and testosterone) as well as activating of multiple signaling pathways (Kero et al. 2000). Excluding a few species, mouse and human adrenal development follows relatively similar steps with elevation of adrenal androgen levels in the prepuberty period in primates (Else and Hammer 2005). The mechanisms underlying this process named adrenarche remain unknown, largely due to the limited availability of experimental models. Since accessible literature is still controversial whether rodents possess or not a postnatal expression of 17-hydroxylase/17, 20-lyase (CYP17A1) definitive cortex cell lines are often applied for studies (Bélanger et al. 1990; Yoshimoto and Auchus 2015; Huhtaniemi et al. 2018). Noteworthy, in human mutation in CYP17A1 results in congenital adrenal hyperplasia (rare inherited autosomal recessive disorders) characterized by an increase in steroidogenic hormone precursors that are mainly converted into androgens (Huynh et al. 2009). In adrenal physiology, relevant gender and age differences in e.g., components of the hypothalamic-pituitary-adrenal axis, adrenal weight, central axis, and adrenal hormone levels were revealed (Bielohuby et al. 2007; El Wakil et al. 2013).

In rodents and humans, the adrenal cortex expresses androgen receptor (AR) (Simental et al. 1991; Trejter et al. 2015) making the adrenal a target of androgen signaling. This receptor is essential for the regression of the X-zone in male mice (Gannon et al. 2021). The AR is a ligand-dependent transcription factor that controls the expression of specific genes responsible primarily for male-specific phenotypes and reproductive physiology, but also female reproductive functions (Chang et al. 2013). In 2014, a member of the zinc and iron-like protein zinc transporter family (ZIP9; SLC39A9) was demonstrated to display specific, high-affinity binding of testosterone (Berg et al. 2014). Up to today, the role of ZIP9 mainly in the male reproductive system has been studied only in some aspects by us and others (Thomas et al. 2014; Bulldan et al. 2016; Kamińska et al. 2020; Profaska-Szymik et al. 2020).

According to Jackson Laboratory Company (Bar Habour, Maine, USA), activating or gain-of-function mutations in mice with constitutive luteinizing hormone receptor (LHR) signaling (KiLHRD^582G^) appear limited to exon 11 of luteinizing hormone receptor gene (Lhcgr). Most of the mutations are clustered in transmembrane helix 6 and the third intracellular loop with aspartic acid at position 578 most commonly mutated to glycine (D578G) that is constitutively active. The active mutation in D582G was confirmed by the cyclic cAMP level that is 23-fold higher in KiLHRD^582G^ when compared to wild-type mice. Expression of genes involved in the steroidogenesis, e.g., Lhcgr, steroid acute regulatory protein (Star), cytochrome P450 side-chain cleavage (Cyp11a1), Cyp17a1, and 3β hydroxysteroid dehydrogenase type V1 (Hsd3b6) is upregulated in Leydig cells. McGee and Narayan (2013) and Hai et al. (2017) demonstrated that constitutive LHR signaling in KiLHRD^582G^ mice causes sexual dysfunction being a model for studying erectile/ejaculatory perturbations and Leydig cell tumorigenesis. Heterozygous KiLHRD^582G^ mice exhibited precocious puberty, Leydig cell hyperplasia, and elevated testosterone levels. They became infertile by about 6 months of age despite normal sperm count and motility. Up to today, nothing is known about the effect of LHR overexpression on adrenal function.

For decades, rabbit (*Oryctolagus cumiculus f. domesticus*) has been often used as a suitable experimental model in diverse studies including adrenalectomy. These studies assist in the interpretation of pathological and/or experimental findings in various mammals. Nevertheless, rabbit adrenal biology still remains not well-studied *per se*. Rabbit adrenal may differ in weight in individuals but there are no gender-specific differences (Stamatova-Yovcheva et al. 2021). Comparative studies showed that the shape, position, and arterial irrigation of the adrenal gland in rabbits are similar to those in rodents (Cardoso da Silva et al. 2020). What is more, in rabbits like in several other species, testosterone-secreting adrenal tumors were described but no studies were undertaken, e.g., on gonad function under these conditions (Lennox and Chitty 2006).

Selenium (Se2+) has high activity as a free radical scavenger and anti-cancer agent also used in diet supplementation (Kieliszek 2019). This trace element is a cofactor of many enzymes, e.g., glutathione peroxidase or thioredoxin reductase. The glutathione peroxidase activity is elevated in the adrenal gland as a consequence of the steroidogenic process. It was found that Se2+ deficiency caused a marked decrease in cellular glutathione peroxidase activity and did not affect basal and short-term adrenocorticotropic hormone-stimulated corticosterone concentration (Chanoine et al. 2004), in light of reports demonstrating a positive role of Se2+ in the function of male reproductive tissues (Shi et al. 2017; Qazi et al. 2019; Duliban et al. 2023). Therefore, it will be exciting to find out whether Se2+ affects androgen signaling in adrenals. On the opinion of the above authors, differentially designed studies and their results can be used to better know some problems in male infertility. According to Dumontet and Martinez (2021), future research exploring adrenal androgen production and signaling will surely help define the physiological role of these hormones in human and non-human primates more precisely. The field of adrenal androgen production remains a crucial yet largely uncharted area of study due to one significant obstacle which is the lack of suitable animal experimental models and systems. Herein, we focused on androgen signaling *via* both canonical and membrane androgen receptors (AR and ZIP9) in the adrenals of transgenic mice with constitutive expression of LHR (KiLHRD^582G^) and these of rabbits subjected to Se2+ treatment. We expect these new insights into central (*via* LH) and local (microelements; Se2+ and zinc interaction) regulation, and relations will help to increase our understanding of the universal and/or species-specific characteristics of androgen adrenal secretion and signaling.

## Materials and methods

### Animals

Male mice 029311; B6129S-Lhcgr^tm1.1Pnara^/J or KiLHR^D582G^ (heterozygous; *n* = 10) were purchased from Jackson Laboratory (Bar Habour, Maine, USA). Male C57BL/6 mice (*n* = 3) were housed in the Animal House of the Institute of Zoology and Biomedical Research, Jagiellonian University in Krakow, Poland. A standard pallet diet (LSM; Motycz, Poland) and water were provided *ad libitum. *Both mice at the age of 5 weeks were euthanized and adrenals were removed. Tissues were fixed in 10% formalin or frozen in liquid nitrogen.

Male rabbits, New Zealand White rabbits x Termond White (*n* = 6) were housed on a farm operated by the Research and Educational Center of the Faculty of Animal Breeding and Biology at the University of Agriculture in Rzaska-Krakow, Poland. Pelleted food (Progress Max, The Haus, Spytkowice, Poland), hay, and water were provided *ad libitum*. The 5-month-old animals were stunned using a penetrating bolt device and after that, the incision of the veins and arteries of the neck was performed according to the Directive of the Ministry of Agriculture and Rural Development from 9 September 2004 and 19 September 2019 based on European Union regulations (Journal of Laws 2019 it. 1966). Both adrenals were removed, and cut in half. The adrenal cortex was isolated under a stereomicroscope, cut into pieces and used for *ex vivo* culture.

Mouse and rabbit breeding, housing, and experimentation were conducted according to the Directive of the European Union Parliament and the Council (2010/63/EU of 22 September 2010) regarding to the protection of animals used for scientific purposes.

The use of animals and all protocols of animal experiments were approved by the Local Ethics Committee in Krakow (permission numbers: 70/2013 and 151/2015).

### Ex vivo cultures and treatment

Rabbit adrenals were cut into small pieces (approx. 2 mm^3^). From the preautoclaved 1.5% agarose, small pillars were prepared a day before the experiment. After solidification, agarose was cut into columns (approx. 8-mm width and 5-mm height). The columns were immersed in the Dulbecco’s modified Eagle medium (DMEM; Sigma-Aldrich; St Louis, MO, USA). Three columns per well were placed into the six-well plates. Tissues were located on top of the pillars (one piece per pillar) in DMEM supplemented with 10% heat fetal bovine serum (FBS) (Sigma-Aldrich) and L-glutamine, 50 U/mL penicillin, 50μg/mL streptomycin (without phenol red with the addition of 5% dextran-coated, charcoal-treated FBS to exclude estrogenic effects caused by the medium). Tissue explants were incubated at 37 °C in an atmosphere containing 95% air and 5% CO_2_. Selenium (Sigma-Aldrich) doses (1, 5, and 10 μg/100 mg tissue) were chosen according to studies by Behne et al. (1996), Shi et al. (2017), and Li et al. (2020). Selenium was dissolved in dimethyl sulfate (DMSO). Stock solutions were shortly stored at −20 °C. The DMSO concentration within the culture medium was < 0.1% (v/v). Control tissues incubated with medium included only the solvent and Se2+-treated tissues were incubated for 48 h.

After incubation, rabbit and mouse adrenal were either used for routine histology (hematoxylin-eosin staining) or frozen tissues were used for western blotting (expression of PCNA, androgen receptors: AR and ZIP9) and testosterone level measurement.

### Cell size measurement

In hematoxylin-eosin-stained adrenal tissue, the diameter of the cortex cells was measured at ×100 magnification using ImageJ software; https://imagej.nih.gov/ij/docs/intro.html. On average, 60 cells were measured per slide. When the cells were slightly oval, only the smaller diameter was measured. The mean was determined for each animal, and data (means ± SD) were expressed in μm.

### Western blot

Separation of protein was performed by SDS-PAGE under reducing conditions and the transfer of proteins to polyvinylidene difluoride membranes. Nonspecific binding sites were blocked with non-fat dry milk containing Tween® 20. Next, the membranes were incubated with the respective primary antibodies anti-PCNA (cat. no SAB2701819 Sigma-Aldrich; 1:500), anti-AR (cat. no. ab74272 Abcam, Cambridge, UK; 1:500), and anti-ZIP9 (cat.no. SAB3500599 Sigma-Aldrich; 1:500) at 4 °C overnight, followed by a horseradish peroxidase-conjugated secondary antibody (Vector Laboratories) at room temperature. Proteins were detected by chemiluminescence and documented with a ChemiDocTM XRS+ System (Bio-Rad Laboratories Hercules, CA, USA). The specificity of antibodies was assessed with the use of positive controls (rodent testes) (not shown). All immunoblots were stripped and re-probed with an anti-β-actin antibody (cat.no. A2228 Sigma-Aldrich; 1:300) as the loading control. The molecular weights of target proteins were estimated by reference to standard proteins (Sigma–Aldrich). To obtain quantitative results, immunoblots were analyzed densitometrically using the ImageLab software (Bio-Rad Laboratories) by an independent observer.

### Testosterone and zinc level measurement

A Testosterone Enzyme Immunoassay Kit (DRG, Inc. Int. Springfield, USA) was used for the measurement of hormone content in adrenal homogenates following the manufacturer’s instructions. No significant cross-reactivity or interference between testosterone and analogs was observed. This assay had high sensitivity (0.083 ng/mL) and excellent specificity for detection of testosterone.

Absorbance (*λ* = 450 nm) was measured with the use of ELISA apparatus (Labtech LT-4500).

The Zinc Quantification Kit (ab102507 Abcam) was used for the measurement of zinc levels in adrenal deproteinized homogenates according to the manufacturer’s protocol. Absorbance was measured at *λ* = 560 nm in the above apparatus.

### Statistics

Each variable was tested by using the Shapiro-Wilk W test for normality. The homogeneity of variance was assessed with Levene’s test or the Brown-Forsythe test. Since the distribution of the variables was normal and the values were homogeneous in variance, all statistical analyses were performed using one-way analysis of the variance (ANOVA), T test, or nonparametric Kruskal-Wallis test, followed by Tukey’s *post hoc* comparison test to determine which values differed significantly from controls. The analysis was made using JASP software (Jeffreys Amazing Statistics Programme). Data were presented as mean ± SD or median ± quartile ranges. Data were considered statistically significant at *P* < 0.05. All experimental measurements were performed in triplicate from material derived from different animals.

## Results

### Morphology and morphometrics of adrenal cortex

**Fig. 1:**
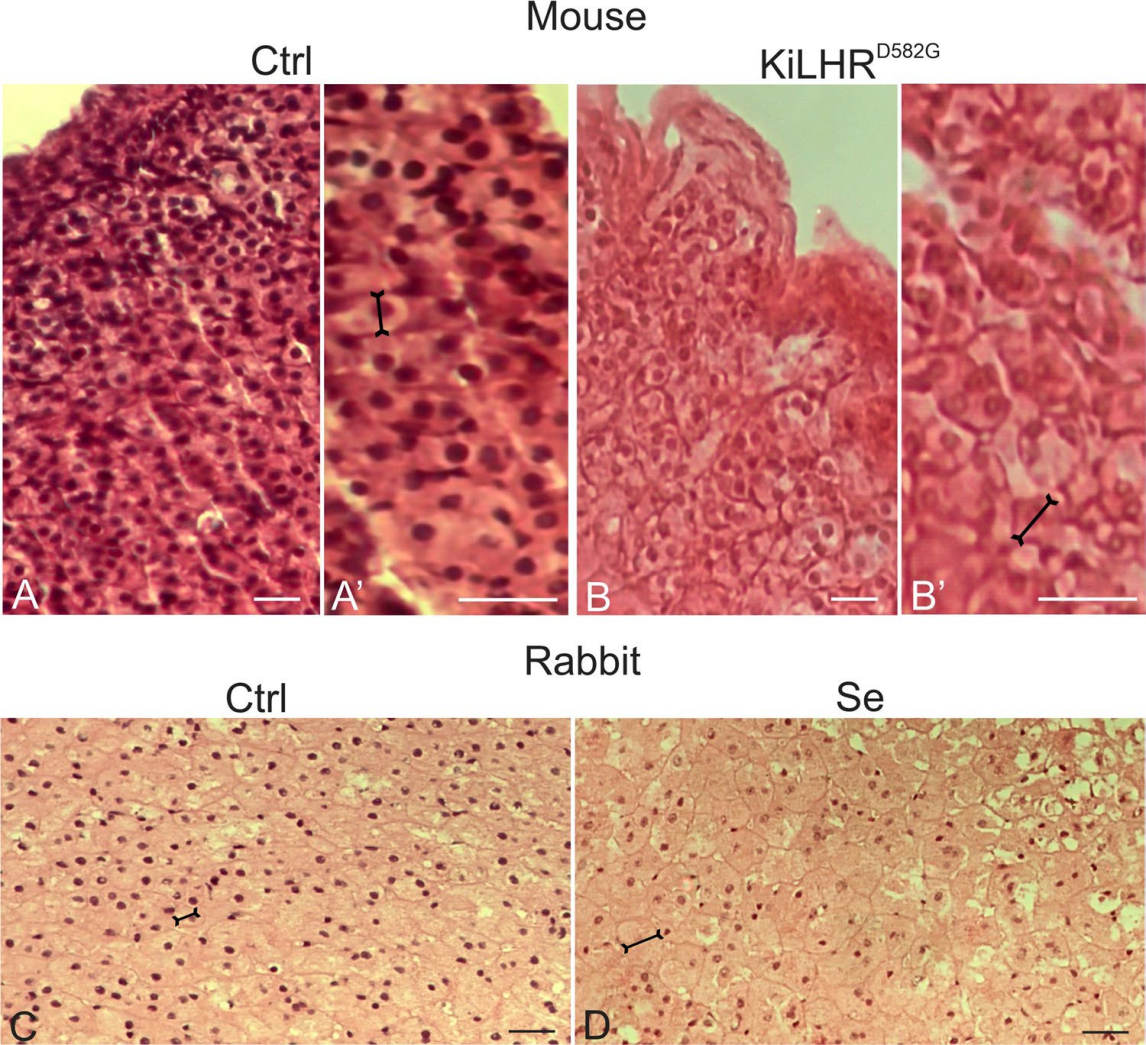
Histological staining (hematoxylin-eosin) of mouse (control vs KiLHRD^582G^) and rabbit (control vs Se2+-treated) adrenal cortex (*zona glomerulosa and/or zona fasciculata*). **A**–**C**—bar 40 μm. **A**′, **B**′—20 μm. Staining was performed at three serial sections from each animal

Compared to the adrenals of control mice, in the adrenal cortex of KiLHRD^582G^ mice, hypertrophy of cells (ZG, ZF and XZ) was observed (Fig. 1A, A′–B, B′). The main diameter of cells increased (17.74 ± 2.66 μm vs 22.69 ± 3.2 μm; *P* < 0.05) (Fig. 1A, A′-B, B′). In rabbit adrenals treated with Se2+ at a dose of 10 μg/100 mg tissue, increased cortex cell size (ZG, ZF and JZ) (36.37 ± 9.08 μm) when referred to control adrenal cortex cell size 27.38 ± 4.86 μm (Fig. 1C, D), and adrenals treated with doses 1 and 5 μg/100 mg tissue, respectively (not shown) were found.

### Expression of PCNA, AR, and ZIP9 in adrenal cortex

**Fig. 2:**
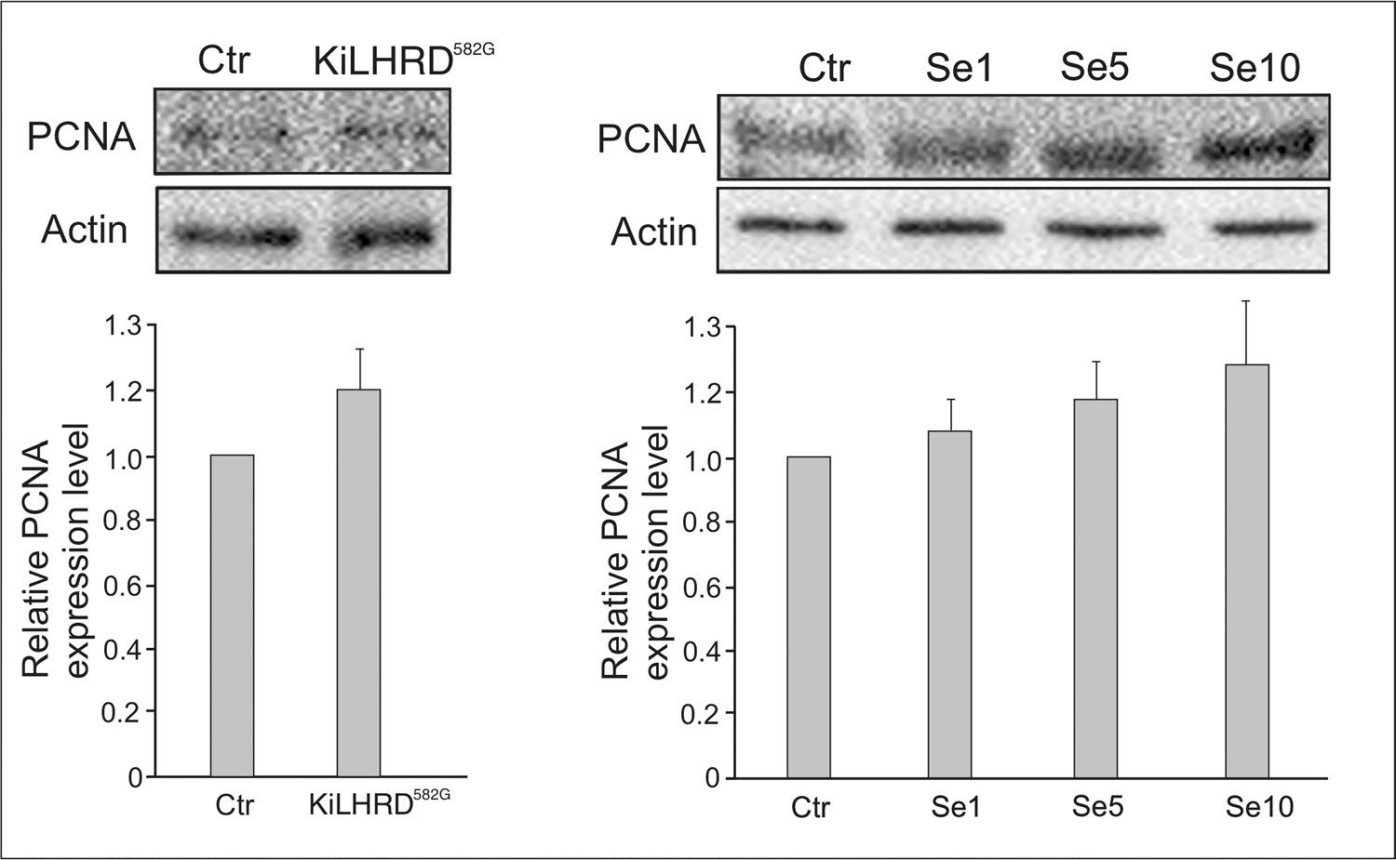
Qualitative expression and relative abundance (arbitrary units) of PCNA in mouse (control vs KiLHRD^582G^) and rabbit (control vs Se2+-treated) adrenal cortex. The relative amount of PCNA normalized to β-actin. The relative intensity of bands from three separate analyses is expressed as means ± SD

**Fig. 3:**
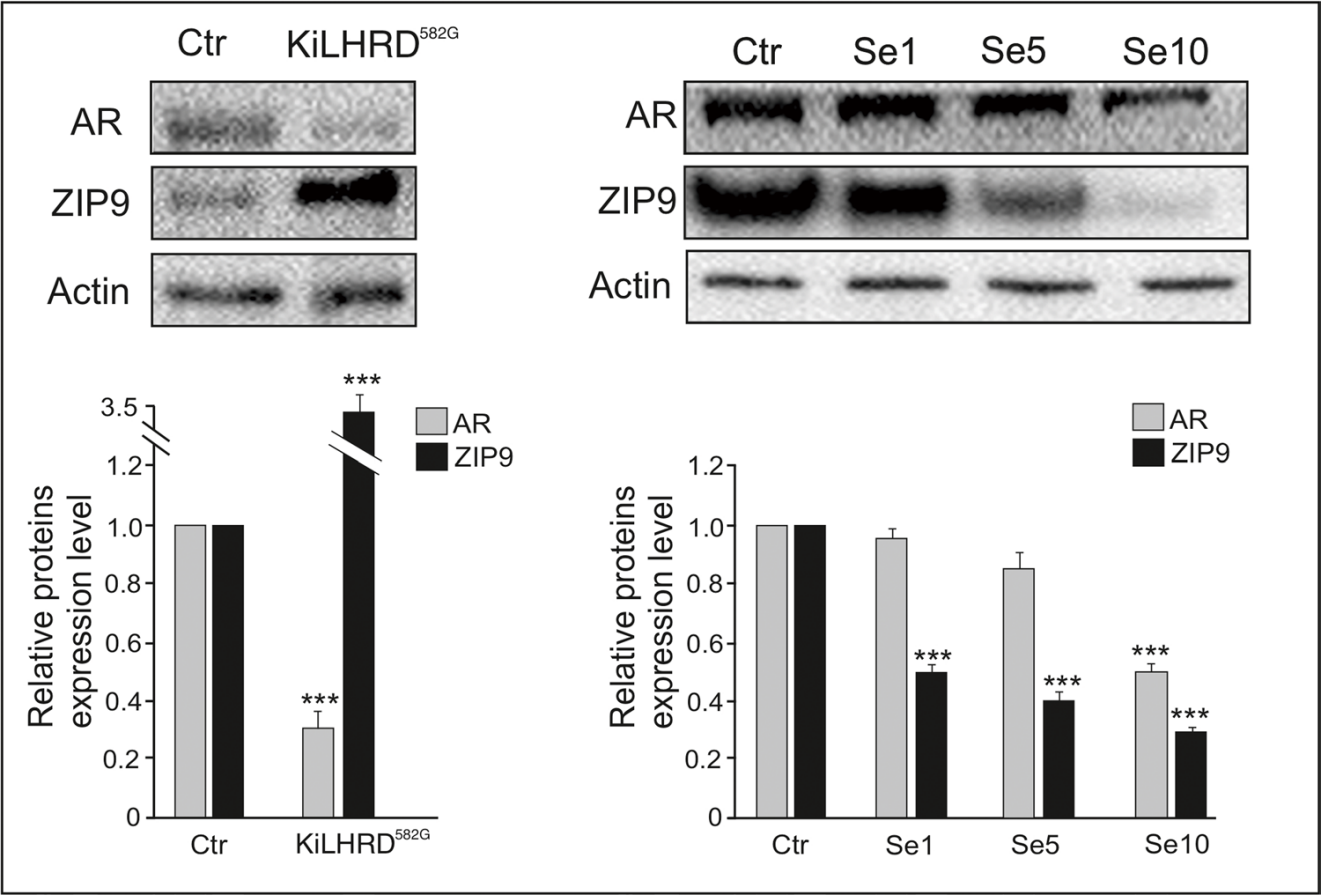
Qualitative expression and relative abundance (arbitrary units) of AR and ZIP9 in mouse (control vs KiLHRD^582G^) and rabbit (control vs Se2+-treated) adrenal cortex. The relative amount of AR and ZIP9 normalized to β-actin. The relative intensity of bands from three separate analyses is expressed as means ± SD. Asterisks indicate significant differences between control and experimental adrenal cortex. Values are denoted as ^∗∗∗^*P* < 0.001

In both KiLHRD^582G^ mouse and rabbit-Se2+-treated adrenal cortex no signs of proliferation were found (expression of PCNA was not changed when compared to respective controls) (Fig. 2). In the adrenal cortex of KiLHRD^582G^ mouse, diverse changes in the expression of both AR and ZIP9 were revealed (Fig. 3). In detail, the expression of AR was significantly decreased (*P* < 0.001) when the expression of ZIP9 was increased (*P* < 0.001). In rabbit adrenal cortex, all doses of Se2+ markedly decreased expression of ZIP9 (*P* < 0.001) while AR expression was affected [decreased; (*P* < 0.001) only by Se2+ at dose 1 μg/100 mg].

### Testosterone and zinc concentration in adrenal cortex

**Fig. 4:**
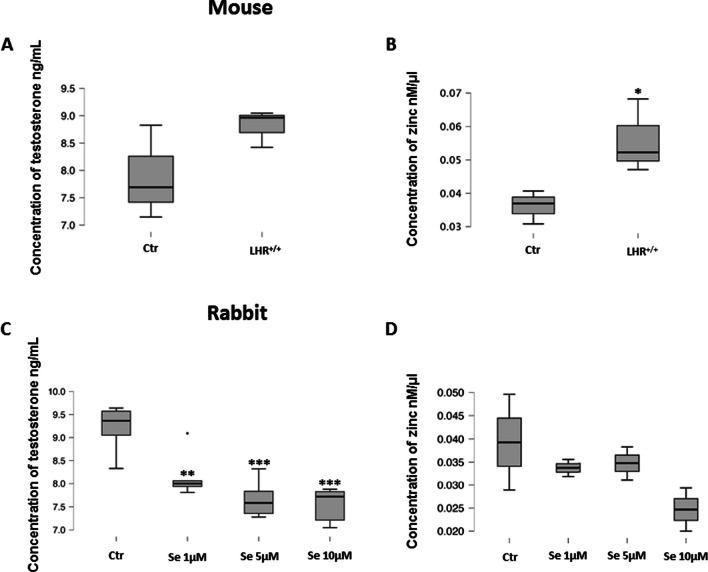
Testosterone and zinc levels in in mouse (control vs KiLHRD^582G^) and rabbit (control vs Se2+-treated) adrenal cortex. Data is expressed as median ± quartile ranges. Asterisks indicate significant differences between control and experimental adrenal cortex. Values are denoted as ^∗^*P *<0.05 and as ^∗∗∗^ *P *<0.001. Analyses were performed in triplicate

In the adrenal cortex of KiLHRD^582G^ mouse, testosterone levels were not changed while a significant decrease (*P* < 0.001) in the levels of this hormone was found in rabbit adrenal cortex treated with different doses of Se2+ (Fig. 4A, C). The increased (*P* < 0.05) zinc concentrations were found in the adrenal cortex of KiLHRD^582G^ mouse while no changes in zinc levels in rabbit adrenal cortex independently of the used dose of Se2+ were detected (Fig. 4B, D).

## Discussion

The phenomenon of adrenal androgen production is currently explained by morphogenes (hormonal influence) (Hornsby 2012). Our herein studies showed, for the first time, that in KiLHRD^*582G*^ mouse, adrenal cortex hypertrophy (ZG, ZF and ZX/ZJ - the latter two zones are the most important in this study) is present together with the unchanged expression of PCNA and the same changes in expression of AR (only by lowest Se2+ dose), but diverse in ZIP9 expression. These may be in part a result of constitutive LHR signaling responding in adrenal androgen production and increased zinc levels including interactions with Se2+ concentration; however, it requires deeper studies. Hypertrophy (considered a non-preneoplastic change) and hyperplasia can often be concurrent lesions in the same gland or even in the same focus (Petterino et al. 2015). To the best of our knowledge, it is the first initial study on the impact of constitutive LHR expression on adrenals. Based on our results in KiLHRD^582G^ mice, the two histological changes as a consequence of constitutive LHR signaling separately exist in the adrenals and testis. This indicates the same mechanism driven by LHR signaling may induce different histological changes. In this study, hypertrophy after Se2+ treatment seems to be based mainly on the interaction of Se2+ and decreased zinc levels with the implication of androgen crosstalk. Dysfunctional adrenal androgen signaling was found to be a possible mechanism in the early development of adrenal hyperplasia in the mouse adrenal cortex (Gannon et al. 2021). Cortical ZF hypertrophy in rodents usually results from elevated levels of ACTH (Lotfi and de Mendonca 2016) while hypertrophy and hyperplasia of ZG can result from derangements of the renin-angiotensin system (McEwan et al. 1996). It is worth noticing here, that in rodent testis, hyperplasia occurs after excessive stimulation of LH or sex steroids (Lakis et al. 2017). On the other hand, it is well known that androgens regulate proliferation in the prostate (benign prostate hyperplasia is also named benign prostatic hypertrophy) (Geck et al. 2000). According to findings showing that over-dosage of zinc intake might increase the risk of visceral adipose tissue hypertrophy (Huang et al. 2017) it is possible that androgen stimulation on ZIP9 can participate in hypertrophy of the adrenal cortex interacting with zinc level, disturbed LHR activity, or extra Se2+ impact. In addition, MacDonald (2000) demonstrated that zinc participates in cell proliferation which is reflected by no changes in PCNA expression in both KiLHRD^582G^ mouse and rabbit Se2+-treated adrenals. Till now, no study has been undertaken to determine the effect of elevated or modulated LH signaling on the adrenal cortex molecular regulation. Here we showed that androgen signaling from adrenals both genomic and membrane-mediated nongenomic are also altered in KiLHRD^*582G*^ mice*. *It seems possible that androgen signaling in adrenals is also implicated in maintaining proper histology and androgen production and action. In addition, we revealed that in case of any disturbances of testis regulation, adrenal histology and androgen production/signaling need to be assessed. It was found by Nishii et al. (2012), that gonadoliberin agonist reduces serum adrenal androgen levels in prostate cancer patients. Based on the Hai et al. (2017) and Hiremath et al. (2020), KiLHRD^582G^ mice exhibit very high levels of testosterone at all postnatal ages, and the supraphysiologic androgen levels are responsible for the loss of muscle in the penile. Huhtaniemi et al. (2018) showed that intact and castrated mouse adrenals synthesize significant amounts of steroids, e.g., testosterone that contribute to the androgen receptor-dependent growth of castration-resistant prostate cancer. Herein, data on testosterone production are in line with the above. In addition, a novel mouse model with a specific ablation of AR in the adrenal cortex (Ad-ARKO) together with a reduction of circulating androgen levels by castration confirmed its usefulness in investigations of androgen signaling in the adrenal cortex. The data demonstrated that AR-signaling is required to protect against adrenal degeneration during aging (Gannon et al. 2019). Hypertrophic changes in XZ, reduction of AR expression, and increased testosterone concentration in KiLHRD^*582G*^ mouse adrenals are in agreement with the results showing an increased adrenal weight and XZ size, despite maintenance of normal testicular testosterone production in Ad-ARKO animals (Gannon et al. 2019). Our results suggest that androgen signaling is finally weakened; thus, it is the one directly responsible factor for adrenal cortex histological changes in KiLHRD^*582G*^ mice. Of note, ZIP9 induces activation of second messengers leading to diverse cellular processes (Taniguchi et al. 2013; Berg et al. 2014; Chen et al. 2020; Profaska-Szymik et al. 2020) including these induced in the absence of androgen stimulation (Converse and Thomas 2020). These processes are not fully discovered in adrenals. It has to be pointed out that ZIP9 controls zinc ion dynamics and homeostasis (Matsuura et al. 2009; Converse and Thomas 2020). This anti-androgen ion was demonstrated to influence the hypothalamic-pituitary-ovarian axis (Jackson et al. 2008). The increase in zinc concentration is consistent with increased ZIP9 expression in the adrenal cortex of KiLHRD^*582G*^ mice indicating ZIP9/zinc effect on histological changes and possible functional changes. What is more, benign prostate hyperplasia and prostate cancer may be associated with a reduction in the levels of tissue and plasma zinc and an increase in urine zinc/creatinine (Zachara et al. 2005; Christudoss et al. 2008). Therefore, it is probable that the interaction of Se2+ and zinc together with testosterone lay upon locally occurring histological and possible functional changes in studied rabbit adrenals *ex vivo.* Talas et al. (2013) reported modulating effects of Se2+ on enzymatic activity and total RNA level in the adrenal medulla of rats exposed to 7,12-dimethylbenz[a]anthracene. In the rabbit adrenal cortex, testosterone production, and ZIP9 were inhibited by a low dose of Se2+, while inhibition of zinc and AR required the highest dose of Se2+. This indicates Se2+ dose-dependent and target-dependent effects. Curiously, after 48 h in culture control, rabbit adrenal testosterone concentration and zinc content appeared to be on the same level as these in wild-type mouse adrenal. Therefore, it is possible that Se2+ (dependently on concentration) affects the central hormonal axis and thus additionally regulates testosterone concentration and signaling. This study sheds some additional light on the central and local regulation, especially with the participation of zinc (Te et al. 2023) and Se2+ (Kong et al. 2011) in testosterone production and action in adrenals. Moreover, further studies both on cellular and subcellular levels are awaited concerning adrenal histo-functional status (e.g., after testis dissection).

**Fig. 5:**
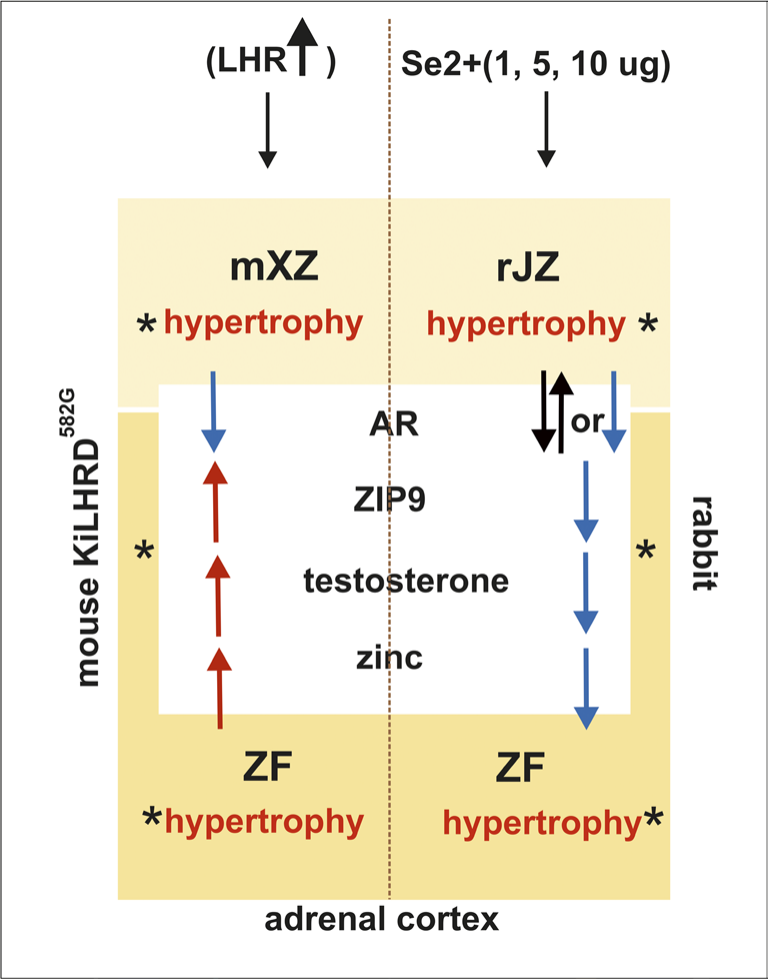
Schematic drawing summarizing obtained results from adrenal cortex of KiLHRD^582G^ mice and adrenal cortex of Se2+-treated rabbit. Arrows indicate changes in studied parameters (indicated by black asterisks: XZ, JZ, and ZF histology and expression of AR, ZIP9 and concentration of testosterone and zinc) in XZ, JZ, and FZ of mouse KiLHRD^582G^ adrenal cortex and rabbit Se2+-treated adrenal cortex: red arrow—increase, blue arrow—decrease, two black arrows—no changes. ZG-*zona glamerulosa* (not shown)*,* ZF-*zona fasciculata*; mXZ - X zone in mouse, rJZ—juxtaordinary zone in rabbit

In summary, (Fig. 5) results obtained by us show that: (i) genetic modulation of LHR signaling reflecting various disturbances of LH action affects adrenal cortex structure (hypertrophy) and function, (ii) excess of Se2+ results in adrenal cortex hypertrophy and functional disturbances, (iii) androgen signaling through AR and ZIP9 may be important regulators of the adrenal cortex in mice and rabbits, (iv) perturbations in AR and ZIP9 signaling in response to LHR overactivity or Se2+ impact are regulated diversely but both may lead to further adrenal tissue pathologies, (v) there is the interplay between androgen signaling *via* AR and ZIP9 as well as Se2+ and zinc which may be implicated in governing of testosterone secretion and action in adrenals including histological structure maintenance, and (vi) mouse (transgenic) and rabbit models, as well as *ex vivo* system, are useful for further adrenal studies.
