# Correction to: Elevated luteinizing hormone receptor signaling or selenium treatment leads to comparable changes in adrenal cortex histology and androgen-AR/ZIP9 signaling

**DOI:** 10.1007/s00709-023-01918-7

**Published:** 2023-12-18

**Authors:** Jaroslaw Wieczorek, Piotr Pawlicki, Marta Zarzycka, Laura Pardyak, Piotr Niedbala, Michal Duliban, Begum Yurdakok-Dikmen, Malgorzata Kotula-Balak

**Affiliations:** 1 Present Address: University Centre of Veterinary Medicine JU-UA, University of Agriculture in Krakow, Mickiewicza 24/28, 30-059 Krakow, Poland; 2 Present Address: Department of Medical Biochemistry, Jagiellonian University Medical College, Krakow, Poland; 3 Present Address: Center of Experimental and Innovative Medicine, University of Agriculture in Kraków, 30-248 Krakow, Poland; 4 Department of Genetics, Animal Breeding and Ethology, Faculty of Animal Science, University of Agriculture in Krakow, Mickiewicza 24/28, 30-059 Krakow, Poland; 5 Department of Endocrinology, Institute of Zoology, Jagiellonian University in Krakow, Gronostajowa 9, 30-387 Krakow, Poland; 6 Department of Pharmacology and Toxicology, Ankara University Faculty of Veterinary Medicine, Dışkapı, 06110 Ankara, Turkey

**Correction to: Protoplasma**

10.1007/s00709-023-01910-1

The correct assigned affiliation of Marta Zarzycka is affiliation 2 and Laura Pardyak is affiliation 3.

The Original article has been corrected.

